# Morphological differences of the reproductive system could be used to predict the optimum *Grapholita molesta* (Busck) control period

**DOI:** 10.1038/s41598-017-08549-y

**Published:** 2017-08-15

**Authors:** Zhiwei Zhang, Lina Men, Yunfei Peng, Jun Li, Angie Deng, Yuan Chen, Xianqian Liu, Ruiyan Ma

**Affiliations:** 1 College of Forestry, Shanxi Agricultural University, Taigu, Shanxi 030801 P.R. China; 2 College of Agriculture, Shanxi Agricultural University, Taigu, Shanxi 030801 P.R. China; 3 Department of Molecular Physiology and Biophysics, Vanderbilt University, Nashville, Tennessee 37232 USA

## Abstract

The oriental fruit moth (OFM), *Grapholita molesta* (Busck), is one of the dominant fruit-boring pests worldwide. In order to conduct integrated control of OFM effectively, it is important to predict the optimum control period. OFM populations have been monitored either by the number of trapped male moths exposed to sex pheromones or by the number of trapped male and female moths using food traps in orchards. The mating status and development stage of the trapped moths have not been characterized. The present paper studies the anatomical morphology of the OFM reproductive system at different development periods. The results revealed that OFM ovarian development can be divided into six stages. The average daily fecundity of OFM had an excellent positive correlation (r = 0.86) with the percentages of OFM in the egg maturation & oviposition stage, which could be used as an indicator in field population prediction work. There were obvious differences in the morphology of the corpus bursa and the heavy muscular area of the ductus ejaculatorius simplex before and after mating, and these differences could be used to increase the accuracy in predicting the optimum OFM control period.

## Introduction

The oriental fruit moth (OFM), *Grapholita molesta* (Busck) (Lepidoptera, Tortricidae), is one of the dominant fruit-boring pests worldwide^1^, and is the most destructive pest in Northern China orchards through its dramatic reduction of fruit quality^2^. In recent years, the damage caused by OFM has worsened because of structural adjustments in the agricultural industry, the increase in cultivated fruit species, the expansion of areas for fruit-planting, the change in cultivation measures, climatic change, etc.^3^. OFM poses a serious threat to many kinds of pome and stone fruits, such as pears, peaches, apples, and plums in the orchards of Northern China^3–6^. It is difficult to control OFM because of their small body size, larval ability to bore into fruits and shoots, host-transforming habits, and large numbers of annual reproductive generations and generation overlap^7^.

In terms of integrated pest control, predicting the optimum control period appears to be very important. Due to difficulties in prediction and control in the past, the prediction of OFM population size mainly relied on the preliminary analysis of trapping numbers of local basic moths exposed to synthetic sex pheromone^8–10^. However, controlling OFM using sex pheromone was only effective for males and in areas where the immigration of gravid females was precluded^11^. Thus, immigration of such gravid females may threaten orchards protected by the mating disruption technique. Food trapping methods that attract OFM females and males effectively complement well-established pheromone-based techniques^12^. The mating status and development stage of trapped moths have not been characterized.

Dissecting and observing ovarian development in female moths was a conventional indicator in forecasting *Pseudaletia* (*Mythimna*) *separata* occurence^13^ and the mating percentage of various age groups in *Heliothis zea* ^14, 15^. Ovarian development is divided into different development levels according to internal structure, such as the maturity level and color of eggs and the consumption of fat^13, 16^. The anatomical morphology and development levels of ovaries can predict the emergence period, moth quantity, and population trend of the investigated Lepidopteran pest^17^, and they could help to determine the optimum period for pest control^13, 16^. Ovarian development is widely used to make predictions about important Lepidopteran pests, such as *Heliothis zes* ^14^, *Pseudaletia* (*Mythimna*) *separate* ^18^, *Ostrinia nubilalis* ^17, 19^, *Cnaphalocrocis medinalis* ^20, 21^, and *Spodoptera exigua* ^22^. These works indicate that analyzing reproductive developmental morphology is an efficient way of predicting the occurrence status of moths. However, reproductive system development and morphological grading based on the different developmental levels of OFM have not been reported, because it is difficult to dissect the small bodies of moths.

In the present report, anatomical morphologies of the reproductive system were carefully studied, and ovarian development was graded according to observed differences in morphology. The correlation between the ovarian development and single female fecundity per day was studied, for it could provide a new, important index in forecasting work. The morphological differences in the reproductive systems of OFM before and after mating were observed as well – they could provide morphological evidence that could increase prediction accuracy and also provide important data for mating behavior research.

## Results

### Anatomical morphology of the reproductive system

We found that the reproductive system of OFM can be divided into two parts, the internal reproductive system and the genitalia. The internal reproductive system includes the gonads, reproductive tract, anterior part of the accessory gland (which growns from the mesoderm), common oviduct, vagina, ductus ejaculatorius, and remaining part of the accessory gland (which grows from the ectoderm). The genitalia include the ovipositor of female adults and the phallus and valva of male adults, all of which grow from the ectoderm^23^.

### Anatomical morphology of the male reproductive system

**Figure 1:**
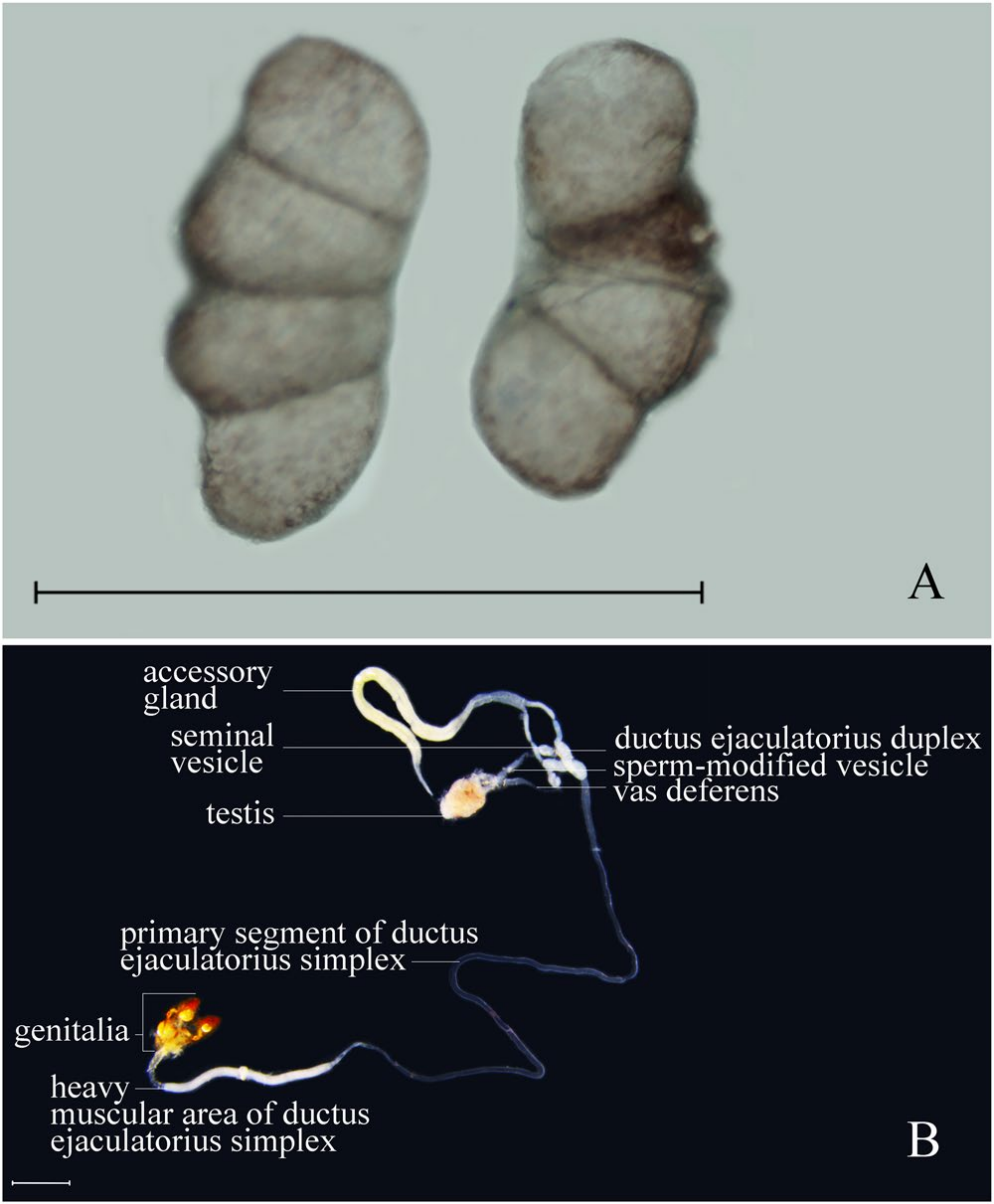
Male reproductive system of OFM. (**A**) Testis of 3rd instar larvae (**B**) Reproductive system of male adults. Scales = 1.0 mm.

Our anatomical morphology analysis showed that the internal reproductive system of male OFM adults includes one testis, a pair of sperm-modified vesicles, a pair of vas deferens, a pair of ductus ejaculatorius duplexes, and one ductus ejaculatorius simplex. Testes are paired into kidney-shaped structures, each sectioned into 4 parts by 3 diaphragm membranes in the larvae and having a milky to light brown color (Fig. 1A). The paired testes merge into a single flat, ellipsoid-shaped structure (one testis) in the late pupae stage and have a light yellow to light brown color in male adults (Fig. 1B). A pair of slender vas deferentia arises from the testis and connects with the ductus ejaculatorius duplex. Each vas deferens has a wide, tubular-shape at basal 2/5 defined as a sperm-modified vesicle and an ellipsoid-shape at distal 1/3 defined as a seminal vesicle. The ductus ejaculatorius duplex connects to the accessory gland anteriorly and to the ductus ejaculatorius simplex posteriorly. The ductus ejaculatorius simplex is about 20.0–24.0 mm long; it bends, folds, and winds in the posterior part of the abdomen and can be divided into two parts: a primary segment and a heavy muscular area. The primary segment is transparent, thin, soft, white, and tubular-shaped. Its length makes up four fifths of the whole ductus ejaculatorius simplex; one end is made of two ductus ejaculatorius duplexes, and the other end is connected with the heavy muscular area. The length of the heavy muscular area makes up one fifth of the whole ductus ejaculatorius simplex and connects with the genitalia – the tube wall increases in thickness, hardness, and diameter.

The accessory gland is located in the anterior part of the reproductive system, about 7.0–9.0 mm in length, composed of two diverticula, and connected to the ductus ejaculatorius duplex by its base. The first 1/8 of the accessory gland’s length does not adhere with the two diverticula. The first quarter of the accessory gland has fewer contents, and the diameter of the remaining 3/4 is wider with greater inner contents. The distal 1/8 length tapers apically. Two diverticula are obviously constricted at the ligation section with the ductus ejaculatorius duplexes (Fig. 1B).

### Morphological development of OFM male reproductive system

Our anatomical morphology analysis showed that differences between developmental stages are mainly represented by testis morphology according to the morphological study of internal reproductive systems in OFM males since other structures of the male reproductive system do not develop completely before emergence. Paired testes of the larvae are situated on both sides of the dorsal blood vessel in the fifth abdominal segment of the third instar larvae. As larvae grow, their testes enlarge gradually. Testes are paired into kidney-shaped structures, each sectioned into 4 parts by 3 diaphragm membranes (Fig. 1A). Right and left testes come into close contact along the middle dorsal line and are enclosed in a single membrane during the male pupae stage. The testis is a single flat ellipsoid-shaped structure in male adults (Fig. 1B). There were no obvious morphological changes in male reproductive systems in correlation with the day ages of unmated males.

### Morphological differences of OFM males before and after mating

**Figure 2:**
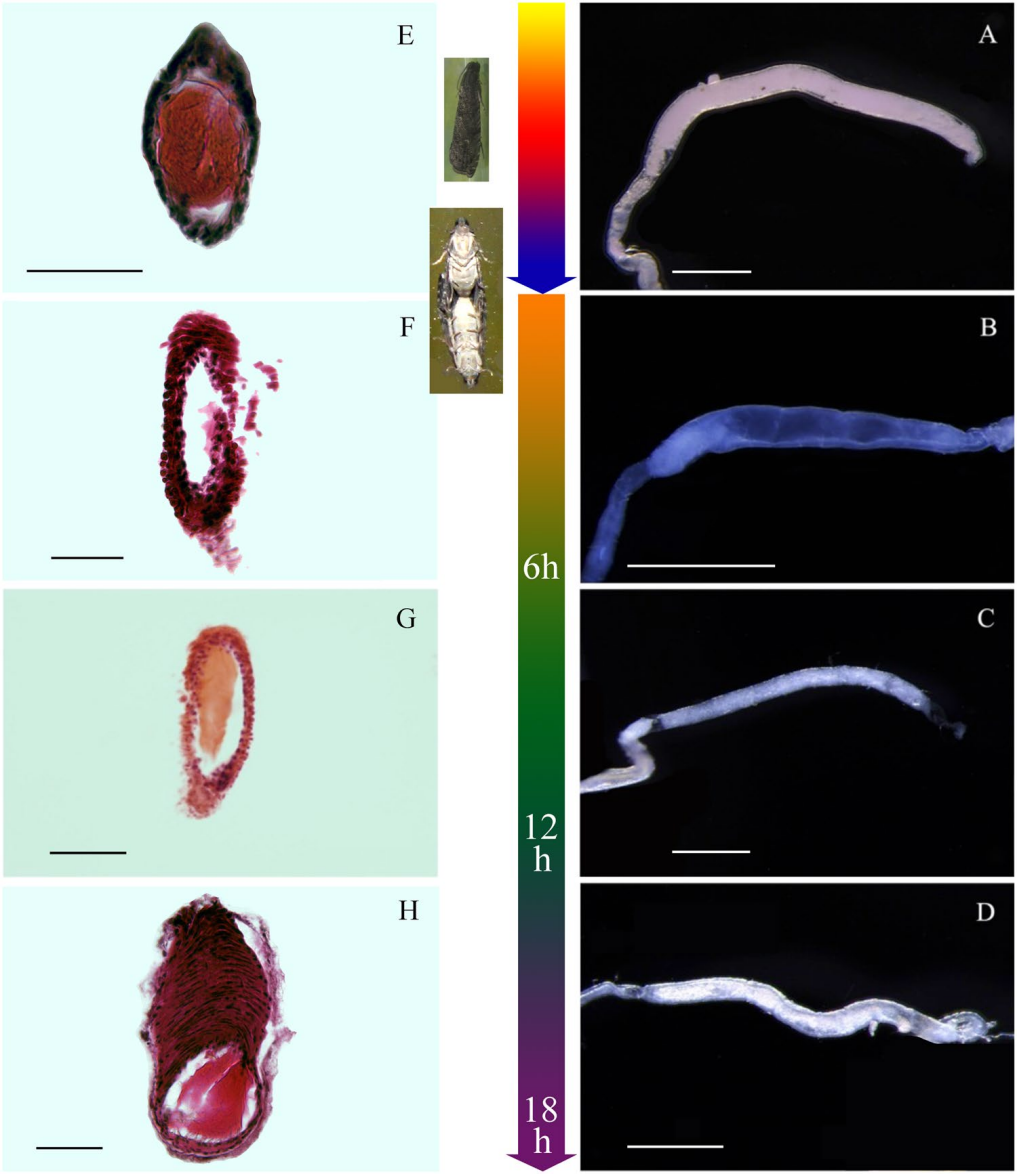
Morphology of the heavy muscular area of the ductus ejaculatorius simplex after mating (h: hours after mating). (**A**) Unmated males; (**B**) 0 to 6 hours after mating; (**C**) 12 hours after mating; (**D**) 18 hours after mating. Scales = 0.50 mm; (**E**) Semithin section of unmated males; (**F**) Semithin section of 6 hours after mating; (**G**) Semithin section of 12 hours after mating; (**H**) Semithin section of 18 hours after mating (oblique view). Scales = 0.050 mm.

We found that the differences between OFM males before and after mating were mainly reflected by different anatomical morphologies of the heavy muscular area of the ductus ejaculatorius simplex. Dense contents were uniformly distributed in the heavy muscular area of the ductus ejaculatorius simplex before males mated (Fig. 2A,E). All of the contents in the heavy muscular area of the ductus ejaculatorius simplex were discharged after mating, and the heavy muscular area of the ductus ejaculatorius simplex was transparent (Fig. 2B,F). The heavy muscular area of the ductus ejaculatorius simplex was still transparent 6 hours after mating (Fig. 2B,F). Some areas of the heavy muscular area of the ductus ejaculatorius simplex had been filled with thin contents 12 hours after mating, and the contents were fewer than those in unmated males (Fig. 2C,G). The heavy muscular area of the ductus ejaculatorius simplex refilled completely, and the degree of filled contents was close to the degree in unmated males until 18 hours after mating. Filled contents were uniformly distributed in the heavy muscular area of the ductus ejaculatorius simplex (Fig. 2D,H). There was an obvious difference in the inner contents of the heavy muscular area of the ductus ejaculatorius simplex before and after mating, and this difference could have applicable value in increasing the accuracy of predicting the optimum OFM control period. However, the inner contents could be refilled over time after mating, so the applicable value is limited. Further anatomical study of female reproductive system morphology is warranted.

### Anatomical morphology of the female reproductive system

**Figure 3:**
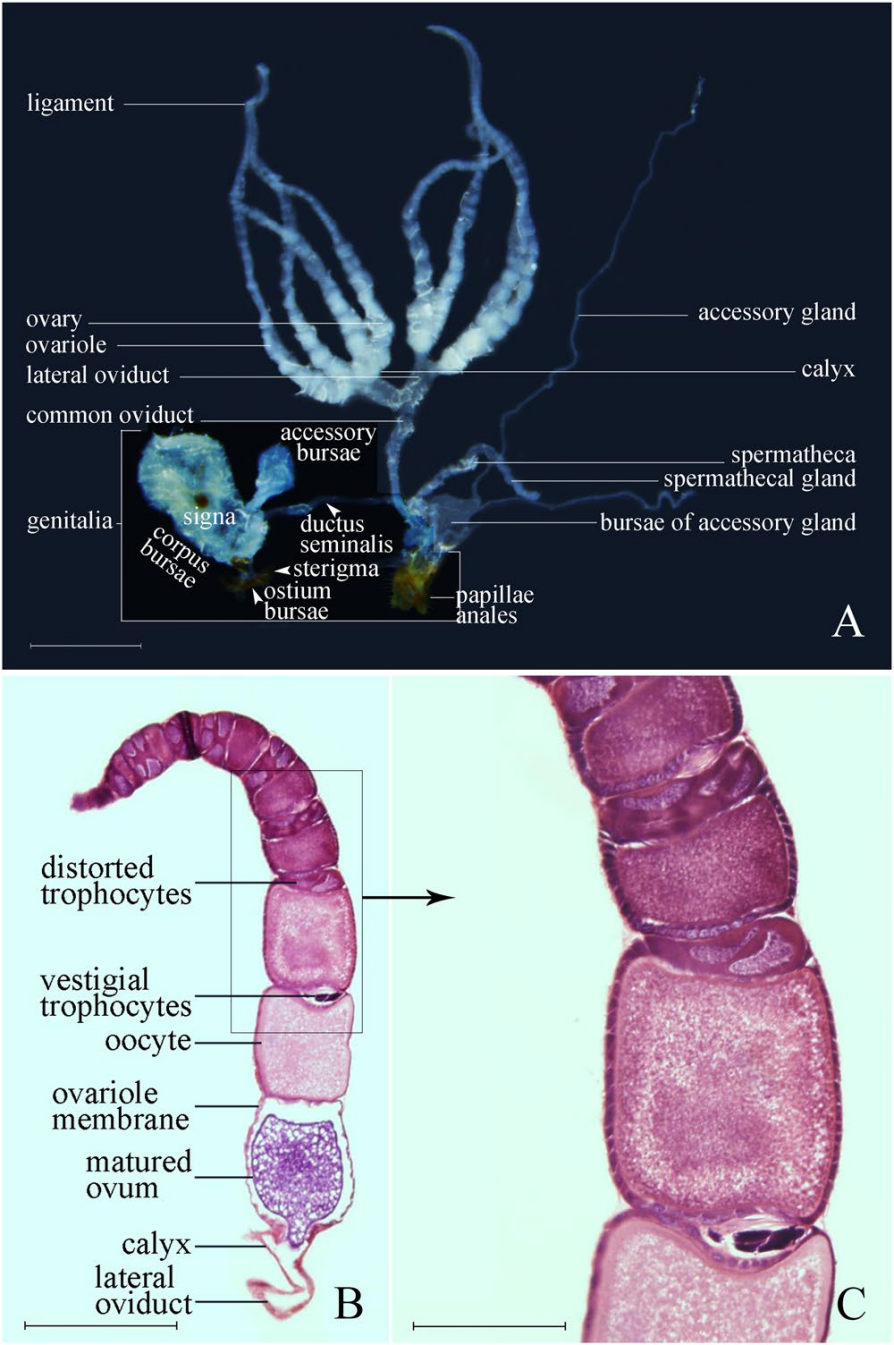
Female reproductive system of OFM. (**A**) Female reproductive system profile. Scale = 1.0 mm; (**B**) Ovarioles semithin longitudinal section. Scale = 0.50 mm; (**C**) Unmatured oocytes and trophocytes in ovarioles. Scale = 0.25 mm.

Our anatomical morphology analysis showed that the internal reproductive system in OFM females, also known as the egg-laying structure, is located in the 3rd to 9th abdominal segments and includes a pair of ovaries, a pair of lateral oviducts, a common oviduct, two accessory glands, and a spermatotheca (Fig. 3A). Each ovary consists of 4 ovarioles, which connect to the calyx of each lateral oviduct (Fig. 3A), and each polytrophic ovariole possesses 7–14 egg chambers (Fig. 3B). Four trophocytes are located between each two oocytes, and trophocytes have become distorted and vestigial with oocyte expansion (Fig. 3B,C). The ovum expands and separates from the ovariole membrane when mature (Fig. 3B). The ligament is gathered together by the terminal filament, which extends from the extremities superior of each ovariole, and the base of all ovarioles merge into one lateral oviduct. The lateral oviducts are thin-walled, slightly inflated, tubular-shaped, and 0.3–0.5 mm in length. Two lateral oviducts merge into one common oviduct in the middle distal part of the abdomen, about 1.0 mm in length. Lateral oviducts and the common oviduct form a pathway for matured ovum discharge (Fig. 3B). The width of the common oviduct is slightly narrower than the lateral oviduct. The posterior part of the common oviduct connects with the genitalia. The spermatotheca arises from the posterior part of the common oviduct, is close to the genitalia, is tubular-shaped, bends and folds, and is about 0.5–0.7 mm in length. One spermatothecal gland is connected with the terminal end of the spermatotheca, blind tubular-shaped, narrower than the spermatotheca, and 5.0–9.0 mm in length. The bursae of the accessory gland, also called the bursae of the collterial gland, connect with the common oviduct at the posterior part of the spermatotheca, is angular-shaped, and has light yellow to brown contents. A pair of accessory glands connects with the terminal end of the bursae of the accessory gland and is slender and long (Fig. 3A).

### Ovarian morphological development of OFM female

#### Six stages of OFM ovarian development

We found that ovarian development begins in the pupal stage after dissecting female OFM larvae, pupae, and adults. The ovarian development of OFM is divided into six stages according to the degree of yolk deposition and the situation of the production and output of mature eggs: namely the initial stage, transparent stage, yolk deposition prophase, yolk deposition stage, egg maturation & oviposition stage, and terminal stage of oviposition.

**Figure 4:**
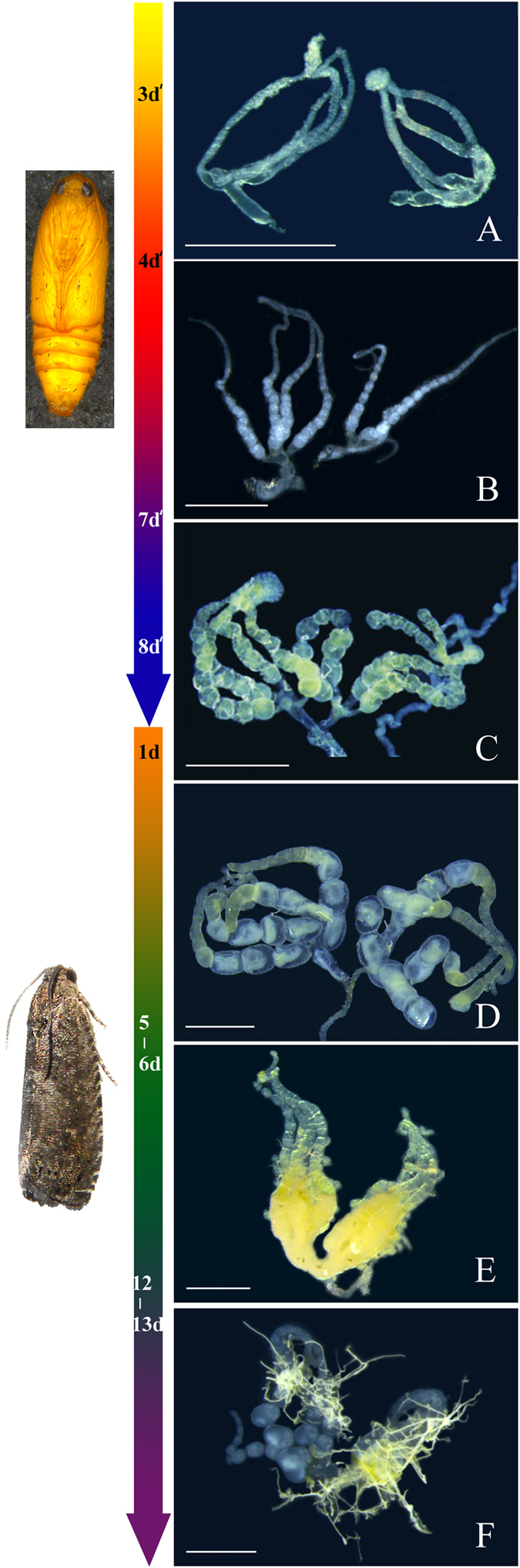
Ovarian classification of OFM females (d’: days after pupation; d: days after emergence). Scales = 1.0 mm. (**A**) Initial stage; (**B**) Transparent stage; (**C**) Yolk deposition prophase; (**D**) Yolk deposition stage; (**E**) Egg maturation & oviposition stage; (**F**) Terminal stage of oviposition.

The first stage is the initial stage. The initial stage occurs 3–4 days after the pupation of female OFM. In this stage, the ovaries begin to develop. Every ovariole is transparent, and no egg-chambers or yolk deposition are present. The tip of each ovary is a mass of tissue: four small ovarioles extend from each organization-mass, and the base of ovarioles is inflated and transparent (Fig. 4A).

The second stage is the transparent stage. Transparent stage occurs 4–7 days after the pupation of female OFM. In this stage, the ovarioles are soft and small, and the ovaries and the oviduct are clearly transparent. Egg-chambers and some transparent, immature eggs in the ovarioles can be found, but none of the eggs have yolk deposition (Fig. 4B).

The third stage is yolk deposition prophase. Yolk deposition prophase occurs 7–8 days after pupation and 24 hours after the emergence of female OFM. In this stage, the ovarioles are longer and thicker than they were in the transparent stage. Some eggs in the ovarioles can be found, and part of the eggs have yolk deposition. However, there are no mature eggs. The epiploa are rich, plump, and spherical (Fig. 4C).

The fourth stage is the yolk deposition stage. Yolk deposition stage occurs 2–6 days after the emergence of female OFM. As ovarioles continue to grow, expand, and mature, separate eggs can be found on the base of the ovarioles. Mature eggs are white, so they can be recognized easily. Yolk deposition occurs in large quantities. The lateral oviduct and common oviduct then begin to expand, neither structure having eggs inside. The number of epiploa slightly decreases, but the morphology of epiploa does not significantly change (Fig. 4D).

The fifth stage is the egg maturation & oviposition stage. This stage occurs 5–13 days after the emergence of female OFM. In this stage, ovarioles are yellowish-white, and the number of mature eggs in the ovarioles increases sharply. The eggs are present in the lateral oviduct and common oviduct, white to yellowish-white, and clearly visible. The yolk deposition of eggs is full. Eggs are closely arranged in the ovarioles; we often observed 3–4 eggs arranged closely in a cylindrical shape. The number of epiploa decreases sharply, and their shape changes from spherical to irregular (Fig. 4E).

The sixth stage is the terminal stage of oviposition. Terminal stage of oviposition occurs 12 days after the emergence of female OFM and ends with their deaths. In this stage, the ovaries began to shrink because of their rapid oviposition of a large number of eggs, and a few mature eggs remain in the ovarioles – some mature eggs become malformed. Nutrition of the female reproduction system is largely consumed, and the shape of the epiploa becomes filamentous (Fig. 4F).

### Ovarian developmental stage of OFM females while mating

**Figure 5:**
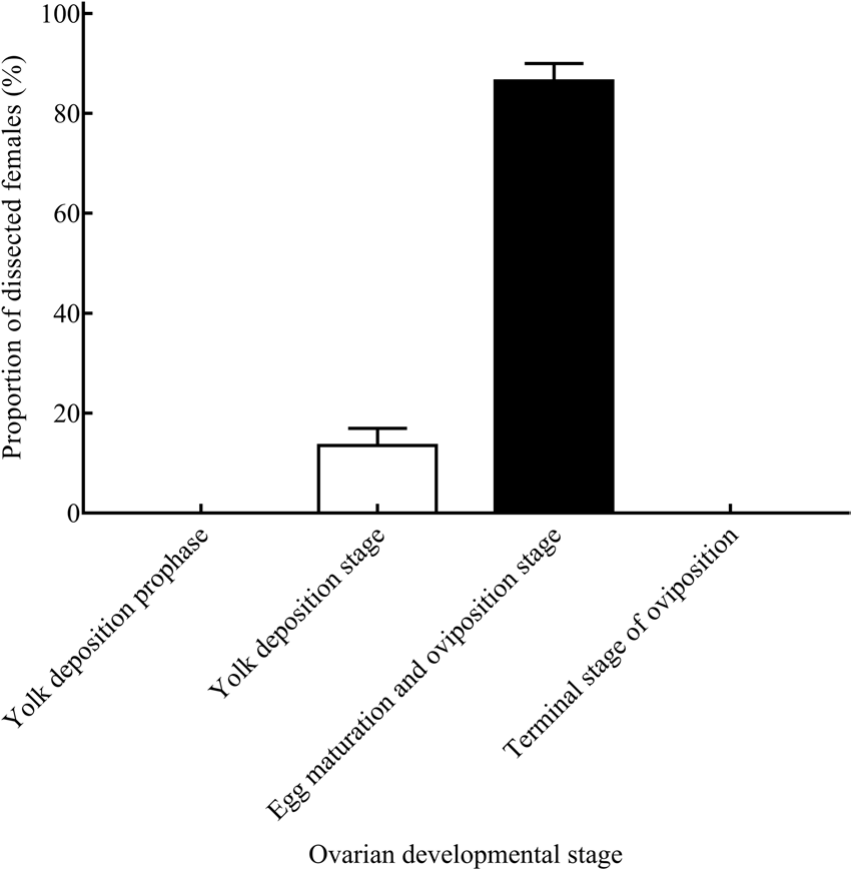
Proportion of female adults (%) in different stages while mating.

The ovarian development stage followed a regular pattern among mating females, so female OFM ovarian developmental stages were best observed when the females were mating. Females were sacrificed while mating, and their ovarian development stages were decided by anatomical morphology directly. 13.50 ± 3.47% were found in the yolk deposition stage, and 86.50 ± 3.47% were found in the egg maturation & oviposition stage. Females belonging to yolk deposition prophase or the terminal stage of oviposition were not found (Fig. 5).

### Relationship between the progress of ovarian development and daily fecundity of the experimental female OFM population

**Figure 6:**
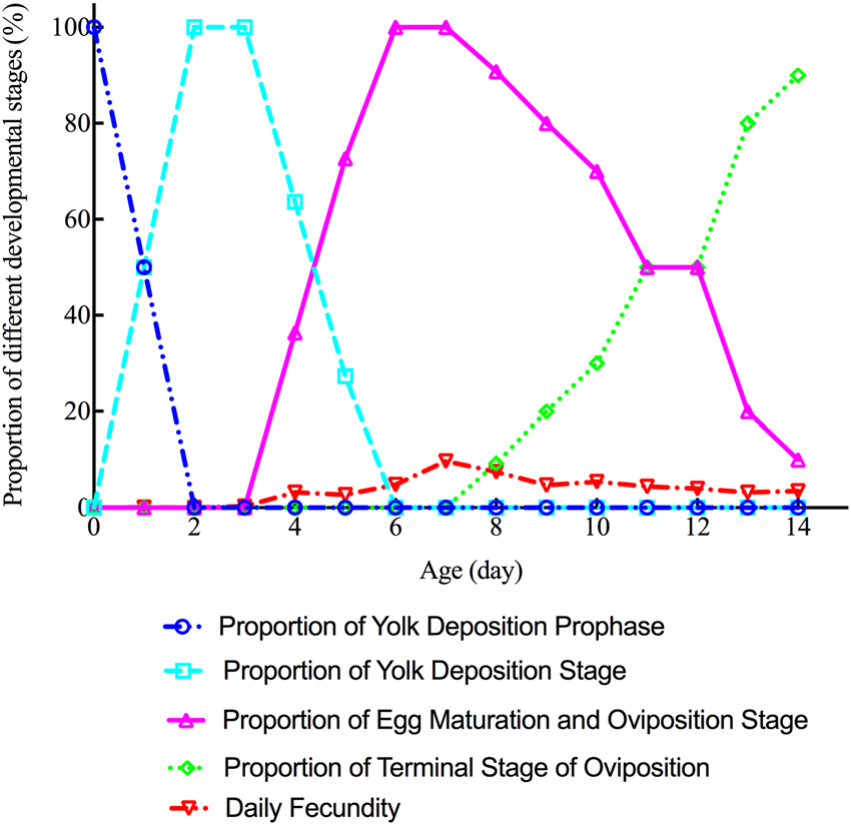
The relationship between daily fecundity and the proportion of OFM females in each ovarian developmental stage.

Data about the daily fecundity of OFM females 15 days after emergence were collected every day. Reproductive system anatomical morphology and the developmental stages of different day age OFM females were acquired. We observed their reproductive system morphology and divided them into ovarian developmental stages. Each ovarian developmental stage of the OFM females was counted (Fig. 6). 100% of females were in yolk deposition prophase after emergence, 50.00% after 1 day, and none after 2 days. However, none of the females were in the yolk deposition stage after emergence; only 50.00% were in the yolk deposition stage after 1 day, and 100% were only observed after 2–3 days. 36.40% of females were in the egg maturation & oviposition stage after 4 days and 100% were after 6–7 days. 50.00% of females were in terminal stage of oviposition after 11–12 days and 80.00% were after 13 days.

**Figure 7:**
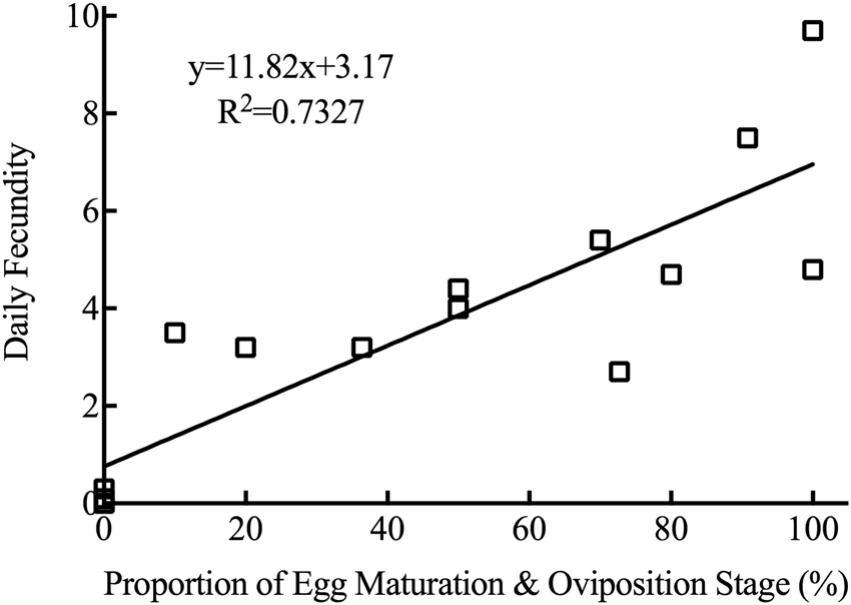
Linear regression model shows that the expectation of daily fecundity is linear to the variable proportion of OFM females in the egg maturation & oviposition stage.

Regression analysis between the progress of ovarian development and the daily fecundity of the experimental OFM population were conducted. The coefficient of determination (R^2^) for the daily fecundity of females in the egg maturation & oviposition stage was 0.7327, whereas the coefficient of determination for the daily fecundity of other stages was less than 0.40 (Table 1). Regression of daily fecundity in the egg maturation & oviposition stage was represented by the equation y = 11.82x + 3.17 (R^2^ = 0.7327). An excellent positive correlation (r = 0.86) was found between daily fecundity and the proportion of females in the egg maturation & oviposition stage as well (Fig. 7).

**Table 1:** Regression analysis of daily fecundity in variable proportion of different ovarian developmental stages of OFM females.

Different ovarian developmental stages	Best-fit equation	Coefficient of determination
Yolk deposition prophase	y = −4.849x + 27.30	R^2^ = 0.2336
Yolk deposition stage	y = −8.268x + 52.22	R^2^ = 0.3823
Egg maturation & oviposition stage	y = 11.82x + 3.17	R^2^ = 0.7327
Terminal stage of ovipositon	y = 1.298x + 17.32	R^2^ = 0.01348

### Morphological differences of OFM females before and after mating

**Figure 8:**
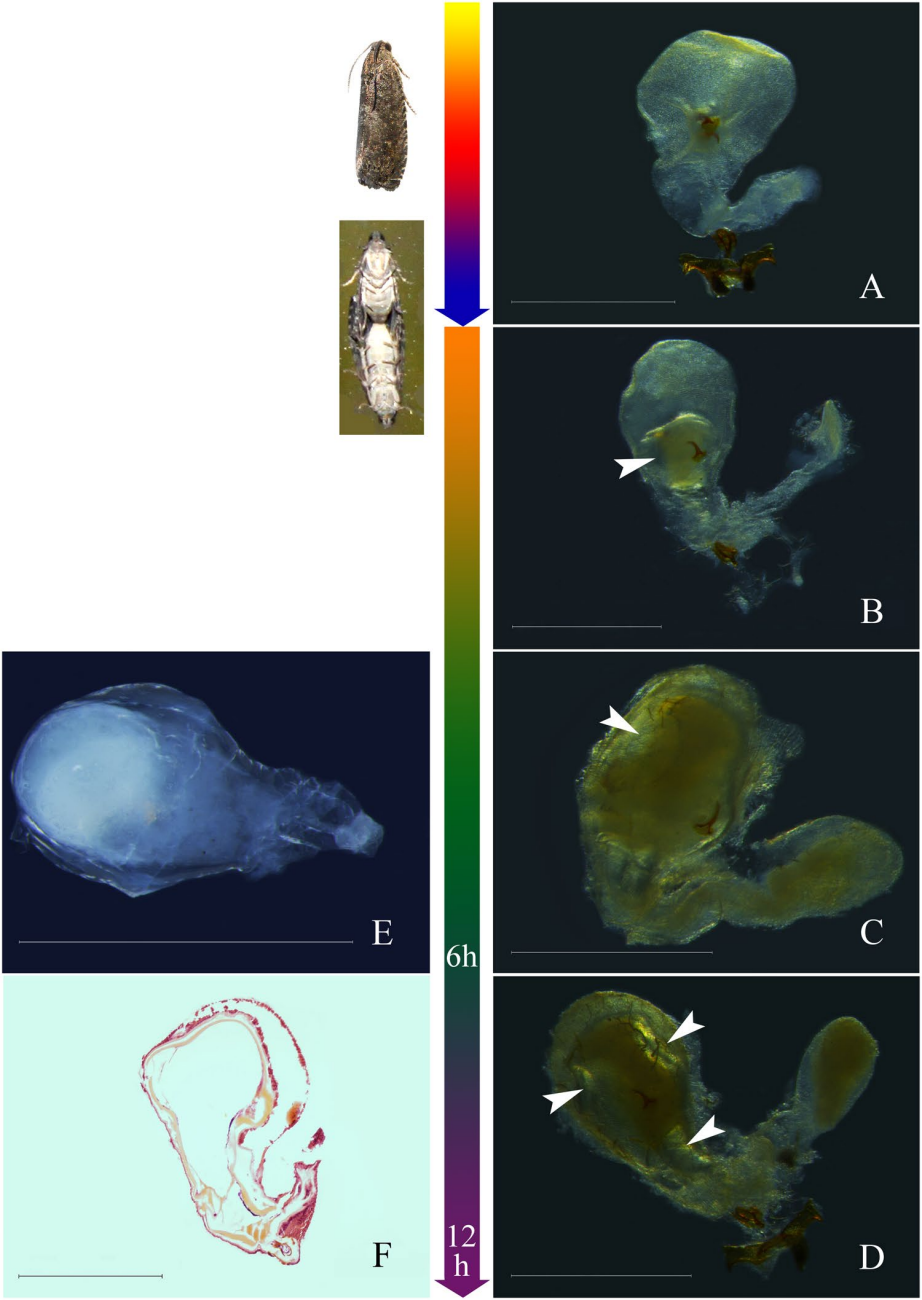
Morphology of corpus bursa before and after mating (h: hours after mating). Scales = 1.0 mm. (**A**) Virgin females; (**B**) Immediately after effective mating (arrow show spermatophore); (**C**) 6 hours after mating (arrow show bubble); (**D**) 12 hours after mating (arrows show bubbles); (**E**) Spermatophore; (**F**) Semithin section of 12 hours after mating.

The different anatomical morphologies of the corpus bursa and the accessory sac of the corpus bursa mainly represented the differences between virgin and mated OFM females. The corpus bursa of virgin females was flat with no spermatophore inside. The accessory sac of the corpus bursa was transparent to translucent, and its contents were thin and transparent (Fig. 8A). The shape of the corpus bursa changed (having a spherical protrusion in the center) immediately after effective mating; there was a white to deep yellow spermatophore in the center of the corpus bursa, while the accessory sac of the corpus bursa remained transparent and did not undergo an obvious change (Fig. 8B). The shape of the corpus bursa did not change obviously 6 hours after mating, but the spermatophore became deformed and expanded/extended towards the accessory sac at the same time. Contents inside the accessory sac increased and appeared light yellow and opaque (Fig. 8C). Contents inside the spermatophore were discharged, and bubbles appeared in the spermatophore within the corpus bursa 12 hours after mating. Contents of the accessory sac increased further, and the color of the accessory sac deepened (Fig. 8D,F). The spermatophore of OFM was not obviously horny, and the external side of the spermatophore was surrounded by a gelatinous substance. The spermatophore was tapered in shape, gradually narrowing towards one rounded apex. Its contents were white to yellowish (Fig. 8E). The morphological changes of the corpus bursa and accessory sac of the corpus bursa were obvious before and after mating (spermatophore absent/present) and this morphological indicator was stable for further application to increase the accuracy of predicting the optimum OFM control period using food trapping.

## Discussion

In the present report, the anatomical morphology of OFM reproductive systems was studied, and the morphological structure of every organ was further confirmed based on previous research^24–27^.

The testis is the site of sperm development and storage in male insect reproductive systems^28^. We found that OFM testes consist of two separate kidney-shaped structures in the larvae and beginning pupal stages, and the testes merge into one spherical structure in the late pupal stage with seminiferous tubules inside (diaphragms were not found in the testes). In morphological studies of male Lepidoptera adults, there is a single testis in the male adult reproductive system of *Pseudaletia separate* ^29^, *Tryporyza incertulas* ^30^, *Choristoneura fumiferana* ^31^, *Buzura suppressaria* ^32^, *Agrotis ypsilon* ^33^, *Ancylis sativa* ^34^, *Pieris melete* ^35^, *Chilo suppressalis* ^36^, and *Colias fieldii* ^37^. However, there are still paired testes in the male reproductive system of *Hepialus baimaensis* ^38^, *Clania variegate* ^39^, and *Antheraea yamamai* ^40, 41^. Testis healing must be based on the specific research taxon, and there are still no obvious similarities in testis structure between OFM and other Lepidoptera species based on previous^27, 30^ and our research, which requires further study. Sperm continue to develop in the seminal vesicle and deposit after being discharged from the testis^42^. The seminal vesicle is ellipsoid-shaped, situated at the distal 1/3 part of the vas deferens in OFM males, and different from many other species of moth^27, 43^. Thus, this structure may be closely related with the volume of the spermatophore and re-mating behavior in moths, though more moth species need to be further studied. The ejaculatory duct includes two parts; the ductus ejaculatorius duplexes and the ductus ejaculatorius simplex, which channels sperm out of the body. The heavy muscular area of the ductus ejaculatorius simplex can temporarily store mature sperm^31^. The heavy muscular area of the ductus ejaculatorius simplex is specialized with two lateral diverticula in *Dioryctria rubella* (Lepidoptera: Pyralidae, Phycitinae)^43^, but there are no diverticula in OFM males. The spermatophore of OFM males have a simple, tapered shape without a particular structure, because the diverticulum of the heavy muscular area of the ductus ejaculatorius simplex probably plays an important role in determining the shape of the spermatophore^44^. The accessory gland consists of two diverticula, and it secretes mucus to provide energy for sperm^45^. Secreted mucus not only bathes and deposits the sperm, but it also plays a role in preventing females from mating again and promotes female spawning^46^. Whether or not anatomical morphology is closely related to the biological function of the accessory gland has not been characterized. Different species affiliated with different Lepidoptera families have two kinds of accessory gland morphologies; one of the two kinds has two diverticula combined partially, as seen in many species belonging to many different families, the other kind has two diverticula dissociated along the whole length, as seen in *Agrotis ypsilon* (Noctuidae), *Buasra suppressaria* (Geometridae), *Mycalesis gotama* (Satyridae), *Pieris rapae* (Pieridae), and *Plutella xylostella* (Plutellidae)^13, 27^. The accessory gland of OFM is organized into the first kind of morphology.

The contents in the heavy muscular area of the ductus ejaculatorius simplex could be used to judge the mating status of *Choristoneura fumiferana*. The contents in the heavy muscular area of the ductus ejaculatorius simplex of unmated male adults were yellow, but they became transparent in the same area 24 hours after mating. The yellow contents were fully restored after 72 hours^31^. *Choristoneura rosaceana* is a similar case, though the restoration time is as long as 6 days^47^. In *Holcocerus hippophaecolus*, the mating status of male adults could be judged by the filled circumstances of the heavy muscular area of the ductus ejaculatorius simplex^48^. Morphological studies found that there are white-to-yellow contents in the heavy muscular area of the ductus ejaculatorius simplex in unmated male OFM adults, but they become transparent in the same area after mating. Double mating and multiple mating behaviors were observed in male OFM adults, and the content refilling process in the heavy muscular area of the ductus ejaculatorius simplex was observed as well. So, we can infer that there is a close relationship between repeated mating behavior and the content refilling process of the heavy muscular area of the ductus ejaculatorius simplex. Contents begin to restore within 6–18 hours and are fully restored after 18 hours. Male adults can mate again after full restoration. There is an obvious difference in the anatomical morphology of the heavy muscular area of the ductus ejaculatorius simplex of OFM males before and after mating, and it could be used to determine the mating status of OFM males. In OFM predicting field works, pheromone-baited traps are often used to monitor pest occurrence. The number of trapped moths is recorded, whereas mating status is ignored. We could increase the accuracy of predictions by determining OFM mating status through morphological observations of the heavy muscular area of the ductus ejaculatorius simplex. The contents of the heavy muscular area are noticeably different before and after mating, so we could use this difference to determine the optimum control period of OFM between pupae emergence and adult mating, since pest control is inefficient in both the larval fruit-boring stage and the period after mating. Guo *et al*. suggest that laboratory OFM populations are preferable over wild populations for conducting experiments. Even after the domestication of over 50 generations of moths, laboratory populations showed better adaptability and greater population growth potential than natural populations (moth life table parameters and damage rates did not decline)^49^.

OFM ovaries are the site of egg formation and development in the female reproductive system. There are two ovaries in female adults, and each ovary consists of 4 polytrophic ovarioles. This morphological character is similar to many other species of Lepidoptera, including many macro- and micro-moths and butterflies^13, 27^, so each ovary consisting of 4 polytrophic ovarioles was a conservative characteristic on the Lepidoptera order level. Lateral oviducts and the common oviduct serve as channels for discharging mature eggs^50^. Oviduct lengths vary among different species, and they were shorter in OFM females than many other species^27^. Spermatophores could be clearly found within female OFM corpus bursae after effective mating. The ductus bursa is tubular, and the spermathecal gland is a single tubular glandular organ in OFM females. The sperm in the spermatophore enters the corpus bursa through the ductus bursa after mating, and secretion from the spermathecal gland can preserve the activity of accepted sperm^51^. The morphology of the ductus bursa and spermathecal gland are less significant to developmental biology and taxonomy research but are vital to reproductive biology research. The accessory gland of moths produces sticky secretions, which can help eggs adhere to other objects^52, 53^. The accessory gland deposits these secretions into its bursae, which are also contractile after egg-laying^54, 55^, so the accessory gland and bursae may be closely related with moth spawning behavior. The shape of accessory gland bursae could be used as an index for judging spawning but not for mating in females. In the present study, the morphology of accessory gland bursae in OFM females has no obvious differences before and after mating.

Ovarian development classification could predict moth occurrence periods^52^, occurrence quantities, occurrence trends, and an optimum control period^3^, and the judgment of mating status^14, 56^ could also increase the accuracy of forecasting. In previous studies of ovarian development of a variety of important agricultural pests in China, such as *Pseudaletia separate* ^29^, *Sogatella furcifera* ^57^, *Agrotis ypsilon* ^33, 58^, *Helicoverpa armigera* ^59, 60^, *Cnaphalocrocis medinalis* ^20, 21^ and *Nilaparvata lugens* ^61^, ovarian development was divided into five stages by different researchers. However, there was no obvious morphological difference between the mature stage and the egg-laying stage in OFM females, so we combined the two stages into the egg maturation & oviposition stage. A total of six stages exist from the beginning of reproductive system moulding in the early pupal stage – there are two ovarian developmental stages during the pupal stage and four ovarian developmental stages after emergence. Yolk deposition of OFM is initiated at the late pupal stage before its emergence, but the duration is short. The results of the four ovarian developmental stages in female adults were similar with other species, such as *Ostrinia furnacalis* ^62^, *Loxostege sticticalis* ^63^, and *Spodoptera exigua* ^22^. Their ovaries began to develop in the pupal stage^64, 65^, yolk deposition appeared before emergence, and the transparent stage did not exist – so ovarian development was divided into four stages^66^. The ovarian development stage of mating females was an important morphological index for the peak mating period, and it could provide vital clues about the best period to control OFM effectively in agricultural practice. The reproductive system morphologies of mating female OFM adults was studied, and we found that the proportion of females in the yolk deposition stage was 13.50 ± 3.47%, and the proportion of females in the egg maturation & oviposition stage was 86.50 ± 3.47% among all studied samples. However, OFM females at yolk deposition prophase or the terminal stage of oviposition were not found; thus, there was no mating during yolk deposition prophase or the terminal stage of oviposition. Yolk deposition prophase is the stage of further ovariole development after emergence, though none of the eggs are mature – there is no mating behavior in this stage. Reproductive nutrition deposited in female reproductive systems was exhausted in the terminal stage of oviposition, so there were no more unfertilized eggs left in the bodies of female adults – no mating behavior in this stage as well. An excellent positive correlation (r = 0.86) was found between daily fecundity and the proportion of OFM females in the egg maturation & oviposition stage, and it could be used as a new indicator to make forecasting data more precise.

Spermatophores within corpus bursa could be used as an index for effective mating status in Lepidoptera^13, 19, 27, 67^, such as *Holcocerus hippophaecolus* ^48^ and *Lampronadata cristata* ^68^. OFM is also in line with this rule. Moreover, spermatophore shape could judge the post-mating period in OFM females. The change in corpus bursa before and after mating is obvious, and their morphology can determine the mating status of OFM females.

## Materials and Methods

### Insects and experimental design

**Figure 9:**
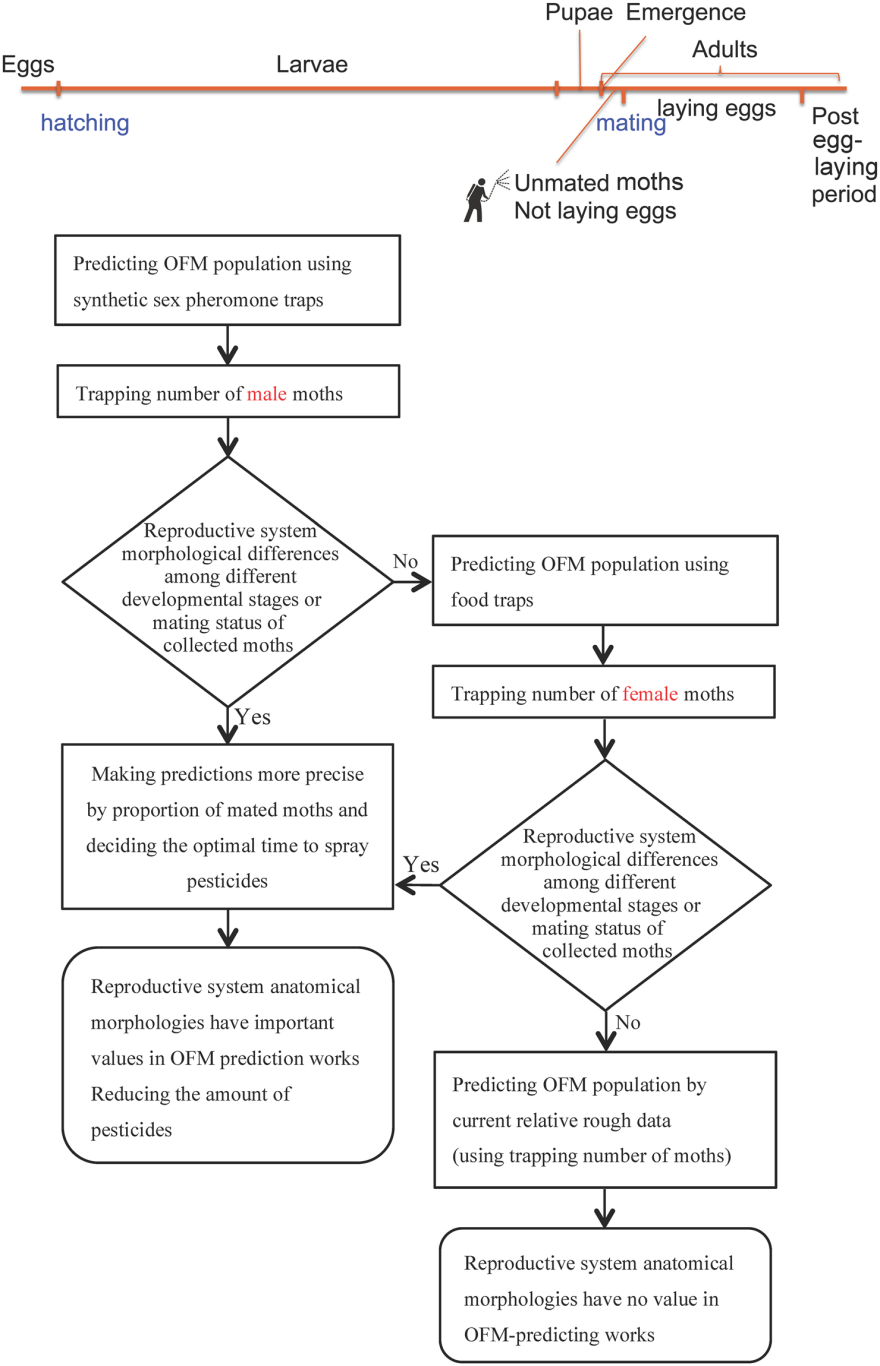
Strategy of predicting the optimum control period for OFM.

The colony of OFM used in this study initiated from caterpillars collected from a peach orchard in Taigu County, Shanxi province, Northern China in the spring of 2008. The larvae were placed in white gauze inside a plastic cage (28 cm in diameter, 12 cm in height) after their removal from host fruits for pupation and emergence. Newly emerged females and males were paired in glass cages (40 cm in diameter, 50 cm in height) covered with absorbent gauze, and 10% (wt: vol) honey water was provided for compensatory nutrition. The inner bottom half of each cage was lined with a piece of sulfuric acid paper for egg collection. The progeny were reared on apples^69^ (*Malus pumila* Miller ‘Gala’) for successive generations in the laboratory under the conditions of 26 ± 0.5 °C, 70–80% RH, and a photoperiod of 15:9 (L: D, lights on at 5:00 am and lights off at 8:00 pm) h in an incubator (SPX-250B-G, Shanghai BoXun, China). They were sub-cultured for more than 30 generations for this experiment^49^. All experimental and feeding conditions were the same. The research strategy of the present report was designed in order to analyze the anatomical morphology differences of the OFM reproductive system and whether or not these differences could be used to increase the accuracy in predicting the optimum OFM control period (Fig. 9).

### Anatomical morphological observation of the reproductive system

After cocooning, thirty male pupae and thirty female pupae were selected randomly in order to dissect and observe the morphology of their reproductive systems every 6 hours until all the pupae emerged.

Unmated males and virgin females were selected randomly to dissect and observe the morphology of their reproductive systems every 6 hours until the moths died naturally. Ten biological replicates (6 individuals were used in each replicate) were conducted for each experimental time point, and the general morphology of the reproductive system was obtained for each experimental time point. Images were acquired by a Leica DFC450 digital camera attached to a Leica stereomicroscope M205C.

#### Paraffin section method

The inner reproductive systems were dissected and fixed in a Bouin’s fluid fixation (saturated aqueous solution of picric acid, 40% aqueous solution of formaldehyde, glacial acetic acid; 15:5:1 v/v), embedded in paraffin, and sectioned into 10-µm thick slices. Sections were double-stained with Hematoxylin and Eosin Stain kit (ScyTek Laboratories Inc. USA) and mounted by Canada balsam (Chemical pure, Tianjin Guangfu Fine Chemical Research Institute, China). The slides were observed and photographed using a DP71 digital camera attached to an Olympus BX61 microscope.

### Morphology under different mating statuses

Newly emerged moths were fed supplementary nutrition, and mating couples were selected randomly at 17:00–19:00^70–72^. Reproductive systems were dissected and studied every 6 hours as soon as selected couples finished mating. The sample number for each developmental stage was at least 30 individuals. Images were acquired by a Leica DFC450 digital camera attached to a Leica stereomicroscope M205C.

### Daily fecundity

The newly emerged moths were fed sup, paired up (male-female ratio of 5:5)^73^, and maintained in another incubator set at the same conditions. Daily fecundity was obtained at 22:00 until all of the adults died.

### Data analysis

DPS (Data Processing System) v15.10 software^74^ and Graphpad Prism 7 (Graphpad Software. San Diego, CA) were used for one-way ANOVA, correlation and regression analysis. Normalized data were transformed using the arcsine square root function before analysis.
